# System of Surgery

**Published:** 1914-09

**Authors:** 


### Reviews of Books.

**System of Surgery.** Edited by C. C. CHOYCE, B.Sc., M.D., F.R.C.S. Pathological Editor: J. Martin Beattie, M.A., M.D., C.M. Vol. III. Pp. xvi, 901. London: Cassell & Co. Ltd. 1914. Price 21s. net.

The present volume is the last of the series. Reviews of the two preceding have already appeared in the pages of this *Journal*. The majority of the articles are extremely good, though a few seem unduly condensed and sketchy in parts.

Special mention must be made of an article by Morriston Davies on the surgery of the lungs and pleuræ.

So much intrathoracic work dates from the time when intratracheal insufflation anæsthesia came into use, that very little can be found about the surgery of the chest in the majority of surgical works.

Mr. Davies gives an excellent account of the operative procedures which have been carried out up to the present. Time alone will show which of these are destined to survive, and to what extent the surgeon will come to the aid of the physician in the treatment of such diseases as pulmonary tuberculosis and bronchiectasis. In the "Surgery of the Cardio-vascular System" will be found the principles which govern the successful suture of vessels. A clear account is also given of the modern surgical treatment of aneurysms.

There is a very useful article by Sherren on injuries and diseases of nerves. It should often prove a handy reference for those surgeons whose memories of anatomy are getting a little rusty. The articles on the brain and spinal cord are among the best in the book. They are admirably compact, lucid and well reasoned. The surgery of the bones and joints is well up to date, and a special article is devoted to the treatment of fractures.

Speaking of the manual as a whole, it may safely be said that it is quite one of the best which has appeared in recent years. It is certainly destined to take its place among our standard works on surgery.

